# Curcumin protection against ultraviolet-induced photo-damage in Hacat cells by regulating nuclear factor erythroid 2-related factor 2

**DOI:** 10.1080/21655979.2021.1994720

**Published:** 2021-12-11

**Authors:** Huiyan Deng, Miaojian Wan, Huaping Li, Quan Chen, Runxiang Li, Bihua Liang, Huilan Zhu

**Affiliations:** a Department of Dermatology, Guangzhou Institute of Dermatology, Guangzhou, China; b Department of Dermatology, The Third Affiliated Hospital of Sun Yat-sen University, Guangzhou, China

**Keywords:** Curcumin, nuclear factor erythroid 2-related factor 2, oxidative stress, photo-damage, skin, ultraviolet

## Abstract

Curcumin suppressed ultraviolet (UV) induced skin carcinogenesis and activated the nuclear factor erythroid 2-related factor 2 (Nrf2) pathway. However, whether curcumin protects skin injury caused by UV is still unknown. A vitro model was established and curcumin effects on Hacat cells were detected. Nrf2 was knocked down in Hacat cells to verify the Nrf2 role in the protective effect of curcumin. Results indicated that ultraviolet A (UVA) (or ultraviolet B (UVB)) irradiation would lead to decreased cell proliferation, increased cell apoptosis, decreased catalase, heme oxygenase 1, and superoxide dismutase expression, and increased levels of protein carbonylation and malondialdehyde (p < 0.05). These adverse events could be reversed by adding 5-μM curcumin. Meanwhile, we found that the application of curcumin effectively induced Nrf2 nuclear accumulation in Hacat cells. While in the Nrf2 knockdown cells, the protective effects of curcumin against UVA (or UVB) were attenuated. Conclusively, curcumin protects Hacat cells against UV exposure-induced photo-damage by regulating Nrf2 expression.

## 1. Introduction

Skin acts as a physiological barrier protecting the body against environmental toxicants, such as pollutants, ionization, and ultraviolet radiation (UV) [1–4]. UVA and UVB, whose wavelengths ranged from 320–400-nm and 280–320-nm, respectively, are harmful to the skin, causing sunburn, erythema, and photoaging. Previous research reported that the skin damage was positively related to the UV irradiation dose and sorts. UVB causes skin lesions of immediate perception and is a high-risk factor for malignant melanoma [5]. Being the major proportion in the solar UV light, UVA penetrates the skin dermis and interacts with skin cells, which further aggravate the destruction of skin tone, elasticity, and wrinkle formation [6]. Lipid peroxidation is an important process that disrupts cell structures and functions by changing membrane lipid structure. As the end product of lipid peroxidation, carbonylation and malondialdehyde (MDA) is considered, a biomarker of oxidative stress, to evaluate the degree of cellular damage and reactive oxygen species (ROS) accumulation [7]. Notably, under oxidative stress, the balance of oxidation and antioxidation is broken, and ROS generated by UV irradiation attacks DNA, proteins, and biomembrane systems, leading to comprehensive cell injury through a series of pathological reactions, such as inflammation, gene mutation, and immunosuppression [8–11]. Hence, it is important to elucidate the relationship between UV irradiation and cascade response triggered by skin injuries.

Nuclear factor erythroid 2-related factor 2 (Nrf2) is a key transcription factor for oxidative stress and activates antioxidant response elements encoded by many cytoprotective genes and antioxidant enzyme genes to control apoptosis, immune, inflammatory, tissue remodeling and fibrosis, carcinogenesis, and metastasis,_by regulating downstream genes, such as thymic stromal lymphopoietin, p38, mitogen-activated protein kinase, and janus kinase 1 [12–17]. Recently, Nrf2 was thought to play a key role in defense of cells against oxidative stress by activating the transcription of a series of antioxidant genes, including phase II detoxifying enzymes, such as superoxide dismutase (SOD), catalase (CAT), and heme oxygenase 1 (HO-1) [18,19]. Furthermore, with the amount of antioxidant effector activation triggered by Nrf2, the organism could be restored from frequent injuries provided by UV light exposure, especially the reduction of ROS formation. M. Schafer et al. have reported that endogenous Nrf2 protects the skin against UV irradiation and plays a crucial role in the maintenance of skin homeostasis. However, the abnormal activation of Nrf2 in a healthy epidermis had an adverse effect on skin integrity [20]. Therefore, it is necessary to validate the role of the Nrf2 signaling pathway in seeking novel strategies for skin injuries.

Recently, curcumin has become a hotspot and is widely applied to various diseases regarding its abundant pharmacological activities, which prohibited anti-proliferative, and apoptosis-inducing effects on different tumor cells [21–25]. As a polyphenol, curcumin could activate kelch-like ECH-associated protein 1-Nrf2-EpRE pathway and display oxidant activity [21,26]. Furthermore, cumulative studies have suggested that curcumin was helpful in treating skin diseases, such as psoriasis, scleroderma, and skin cancer [27–30]. Curcumin suppressed UV-induced skin carcinogenesis by alleviating UVB radiation-induced acute inflammation and photoaging [5]. Additionally, curcumin was thought to play a key role in antioxidant protection due to its ability to activate the Nrf2 pathway and induce phase II cytoprotective enzymes [5]. Regardless, there is no exact interpretation of curcumin effects and mechanisms in UVA and UVB-induced skin injuries.

Therefore, we speculated that curcumin could protect from the UVA and UVB-induced skin injuries through the Nrf2 pathway. This study validates this hypothesis through a vitro model. Curcumin protective effect against UVA (or UVB) irradiation-induced damage in immortalized nontumorigenic human keratinocytes (Hacat) was investigated, then the role of Nrf2 on curcumin exerting photoprotective effects through the interference of Nrf2 expression was validated.

## 2. Materials and methods

### 2.1 Cell culture

Hacat cell lines were purchased from the American Type Culture Collection (Manassas, VA, USA) and grown in Dulbecco’s Modified Eagle’s Medium (Invitrogen, Carlsbad, CA, USA) supplemented with 10% fetal bovine serum (Invitrogen), 1-mM L-glutamine (Invitrogen), 100-U/mL penicillin (Invitrogen), and 100-U/mL streptomycin (Sigma-Aldrich, St. Louis, MO, USA). The incubation condition was at 37°C in a humidified atmosphere containing 5% CO_2_.

### 2.2 Cell viability

The cell viability of Hacat cells was assessed by 3-(4,5-dimethylthiazol-2-yl)-5-(3-carboxymethoxyphenyl)-2-(4-sulfophenyl)-2 H-tetrazolium (MTS) assay (Beyotime Biotechnology, Shanghai, China). The experiment protocol was conducted following previous studies with few modifications [31]. In brief, cells were seeded into 24-well plates (6 × 10^3^ cells per well) for 24 h. Then, cells were pretreated with different curcumin concentrations (Aladdin, Shanghai, China) for 24 h followed with or without UV irradiation. MTS solution was added after 24 h, and the absorbance was observed at 490 nM on Bio-Tek Instruments (Winooski, VT, USA).

### 2.3 Construction of shNrf2-Hacat and NC Hacat

The shRNA target for Nrf2 was synthesized and cloned into the red fluorescent protein (RFP)-containing pBluescript SK (+) plasmid (pU6) followed with a lentivirus package in Genepharma (Shanghai, China). They also provided the negative control (NC) lentivirus. Then, Hacat cells were infected with lentivirus according to the instructions provided by Genepharma. To confirm whether we have got shNrf2-Hacat and NC Hacat, we observed the RFP under an inverted microscope NBI1000 (Siner, Nanjing, China) and collected cells for quantitative real-time polymerase-chain reaction (qPCR) analysis of the Nrf2 expression.

### 2.4 UV radiations

The Hacat cells were washed using hank’s balanced salt solution twice and were exposed to different doses of UVA and UVB irradiation. UVA doses were 2.5, 5, 10, 20, and 40-J/cm^2,^ and the UVB doses were 11.4, 22.8, 34.2, 57, and 79.8-mJ/cm^2^. UVA and UVB instrument models were SS-01 UV light therapy instrument (SS-01A-2) and SS-01 UV light therapy instrument (SS-01B-2), respectively. The SS-01A-2 or SS-01B-2 instrument was preheated for 30 min, then cell culture medium was aspirated and changed to phosphate-buffered saline (PBS), and irradiation distance was 20 cm. UVA and UVB irradiation time were set following the manufacture’s instructions SS-01A-2 and SS-01B-2, respectively.

### 2.5 Cell apoptosis analysis

After exposure to specialized irritation conditions, Hacat cells were washed twice with PBS and then digested by adding 1-ml 0.25% trypsin without ethylene diamine tetraacetic acid. Cells were collected and washed twice with 1× binding buffer in the cell apoptosis kit (Invitrogen). After that, 5-μL allophycocyanin Annexin V and 5-μL 7-AAD were added to 100-μL 1 × binding buffer containing 1 × 10^6^ cells. They were incubated for 15 min in the dark at 37°C. Then, 400-μL 1 × binding buffer was added, and data on apoptosis were analyzed by Novocyte 2040 R flow cytometer (ACEA Biosciences, Hangzhou, China).

### 2.6 qPCR analysis

Total RNA was isolated from cells using RNeasy mini kit (Qiagen, Hilden, Germany) following the manufacturer’s instructions and reverse transcribed to complementary DNA (cDNA) using the iScript^TM^ cDNA synthesis kit (Bio-Rad, Hercules, CA, USA). The target genes and internal reference expression were detected using Taq Pro Universal SYBR qPCR Master Mix (Vazyme, Jiangsu, China) on Step-One PlusTM Real-Time PCR System (Foster city, CA, USA) with specific primers (Qiagen). β-actin was selected to be the internal reference. The primers sequence was listed in Table S1.

### 2.7 Western blotting

Protein was prepared using NEPER Nuclear and Cytoplasmic Extraction Reagents Kit (Thermo Fisher Scientific, Waltham, MA, USA). The protein concentration was determined using a BCA Protein Assay kit (Thermo Fisher Scientific). An equal amount of total proteins was separated on sodium dodecyl sulfate, sodium salt polyacrylamide gel electrophoresis and then were transferred onto a polyvinylidene fluoride (PVDF) membrane (Millipore, Bedford, MA, USA). Then, the PVDF membrane was incubated in 5% nonfat milk dissolved in tris-buffered saline wash buffer with 0.05% Tween 20 (TBST). Subsequently, the membrane was incubated with primary antibodies overnight at 4°C followed by incubating horseradish peroxidase-conjugated secondary antibody at 37°C for two hours. Finally, an ECL detection kit (Millipore) was used to detect the protein-antibody complexes. The primary antibodies were NRF2 (dilution 1:1,000; #ab137550; Abcam, Cambridge, MA, USA), histone H3 (dilution 1:1000; #HL102-01; TransGen Biotech, Beijing, China) and GAPDH (dilution 1: 1,000; #ab9485; Abcam). ImageJ software (National Institutes of Health, Bethesda, MD, USA) was used for protein quantification. The ratio of nuclear protein (NER)/cytoplasm protein (CER) was equal to (NER of Nrf2/H3)/(CER of Nrf2/GAPDH). Then, the relative ratio of NER/CER was calculated compared to the control or negative control (NC) group.

### 2.8 Protein carbonylation and MDA assay

Protein carbonylation and MDA expression were determined using the protein carbonylation kit (Jiancheng Bioengineer Institution, Nanjing, China) and MDA Assay Kit (Beyotime) according to the manufacturer’s protocol.

### 2.9 Statistical analysis

Experiments were conducted at least thrice, and results were expressed as mean ± standard deviation. Data analysis were conducted using SPSS 22.0 software. (IBM, Armonk, NY, USA). Statistical significance was determined using t-test or one-way analysis of variance. P < 0.05 was considered statistically significant.

## 3. Results

Curcumin suppressed UV-induced skin carcinogenesis and activated Nrf2 pathway, and therefore we speculated that curcumin could protect from the UVA and UVB-induced skin injuries through the Nrf2 pathway. In this study, we constructed a in vitro model and then tested curcumin effects on UV-induced Hacat cells. Additionally, Nrf2 was knocked down in Hacat cells to verify the Nrf2 role in the protective effect of curcumin. Results indicated that UVA or UVB irradiation would lead to decreased cell proliferation, increased cell apoptosis, decreased CAT, HO-1, and SOD expression, and increased levels of protein carbonylation and MDA. These adverse events could be reversed by adding 5-μM curcumin. Meanwhile, application of curcumin effectively induced Nrf2 nuclear accumulation in Hacat cells. While in the Nrf2 knockdown cells, the protective effects of curcumin against UVA (or UVB) were attenuated. In conclusion, curcumin protects Hacat cells against UV exposure-induced photo-damage by regulating Nrf2 expression.

### 3.1 UV irradiation-induced damage in Hacat cells

**Figure 1.**
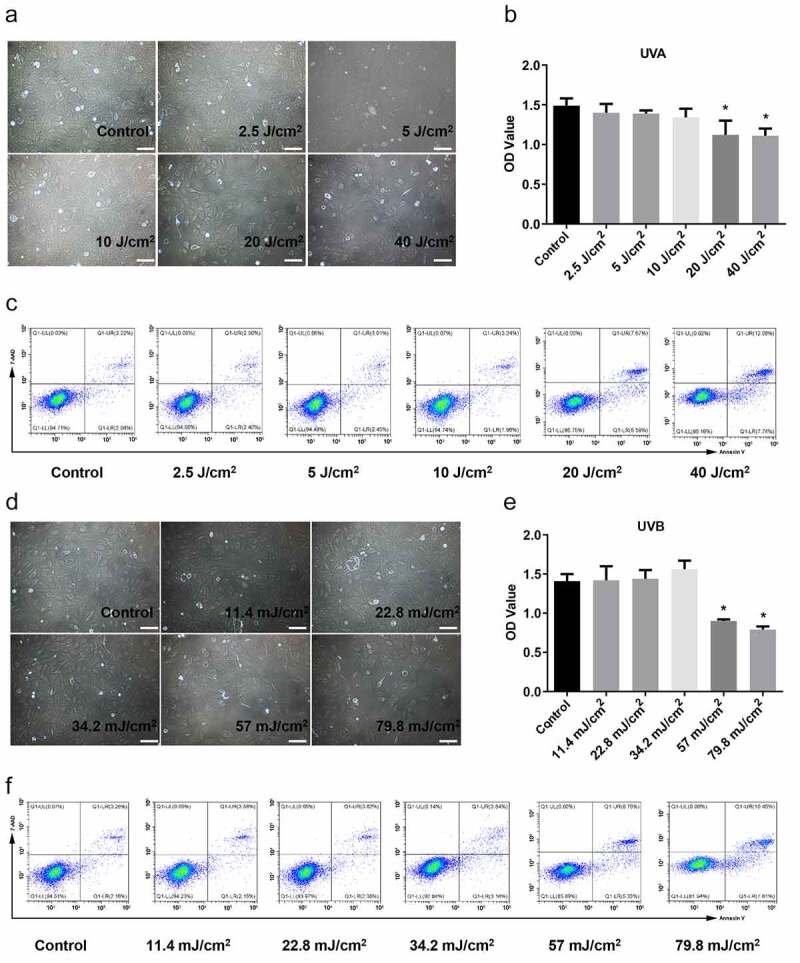
UV irradiation-induced damage in Hacat cells. (a) Morphology of Hacat cells under the exposure of 0, 2.5, 5, 10, 20, and 40 J/cm^2^ doses UVA, respectively. Magnification: 200 × . (b) The OD values of Hacat cells with different does UVA irradiation. (c) The proportion of apoptotic cells in different dose UVA irradiation groups. (d) The morphology of Hacat cells under exposure to 0, 11.4, 22.8, 34.2, 57, and 79.8 mJ/cm^2^ dose UVB. (e) The OD values of Hacat cells with different dose UVB irradiation. (f) The proportion of apoptotic cells in different does UVB irradiation groups. (UVA: ultraviolet A; UVB: ultraviolet B; *p < 0.05, compared to the control group)

A photo-damage model in Hacat cells was established by irradiation with UVA dose (2.5, 5, 10, 20, and 40-J/ cm^2^) and UVB dose (11.4, 22.8, 34.2, 57, and 79.8-mJ/cm^2^). Results indicated no significant change in the morphology of Hacat cells under the exposure of 2.5–10-J/cm^2^ dose UVA (Figure 1a). While cells were deformed, shrank in volume, and karyopyknosis after exposing 20 and 40-J/cm^2^ dose UVA irradiation, with increased cell debris and necrosis (Figure 1a). Cell viability analysis (Figure 1b) showed that the damage degree gradually aggreviated with increased UVA irradiation dose, and 20-J/cm^2^ or more of UVA irradiation damaged cell survival (p < 0.05). The apoptotic and necrotic cells were detected by flow cytometry analysis after using 7-AAD and Annexin V double staining. The proportion of early apoptotic cells (7-AAD^−^ and Annexin V^+^), late apoptotic cells (7-AAD^+^ and Annexin V^+^), and necrotic cells (7-AAD^+^ and Annexin V^−^) were different in various groups. The total proportion in aforementioned three regions is more than 10%, suggesting that Hacat cells were significantly damaged. The results showed that the apoptosis and necrotic rate in 20-J/cm^2^ and 40-J/cm^2^ UVA groups were 13.25% and 19.82%, respectively, indicating significant damage (Figure 1c). Similarly, the extent of damaged cells correlated with UVB irradiation intensity (Figure 1d). The cell viability and apoptosis analysis showed that significant apoptosis occurred in 57-mJ/cm^2^ and 79.8-mJ/cm^2^ UVB groups (Figure 1e and 1f). Consequently, we suggested that the Hacat cells were damaged by exposing more than 20-J/cm^2^ UVA or more than 57-mJ/cm^2^ UVB. Therefore, we chose the UVA dose of 20-J/cm^2^ and UVB dose of 57-mJ/cm^2^ for the following experiments.

### 3.2 Investigation of the proper concentration of curcumin

**Figure 2.**
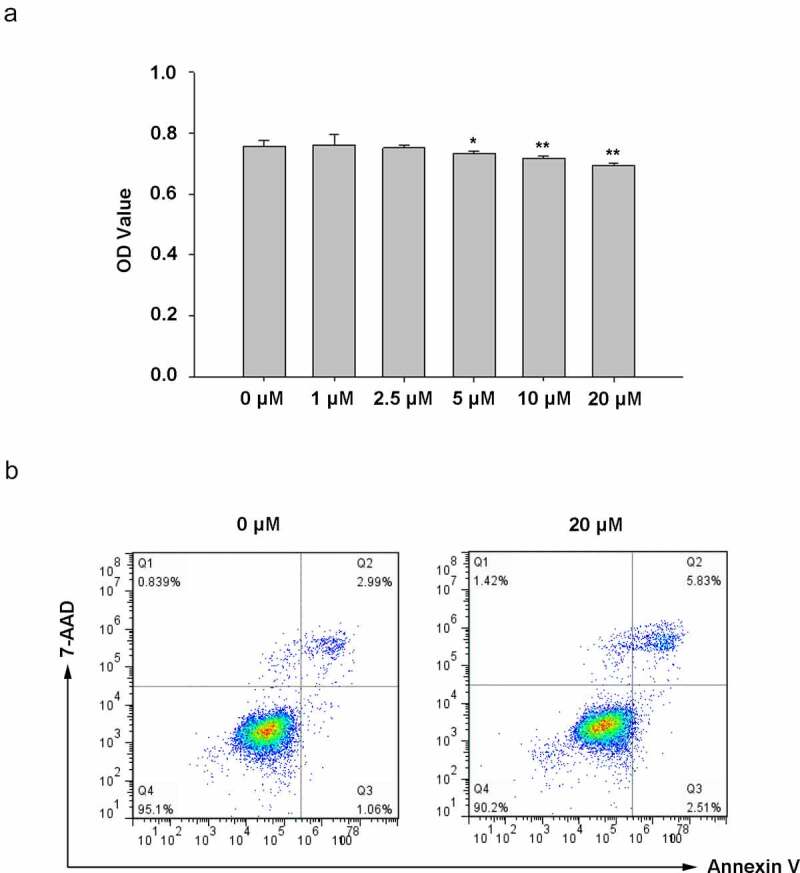
The proper concentration of curcumin selection. The proliferation (a) and apoptosis (b) of Hacat cells in different concentrations of curcumin-treated groups. (*p < 0.05, **p < 0.01, compared to the control group)

**Figure 3.**
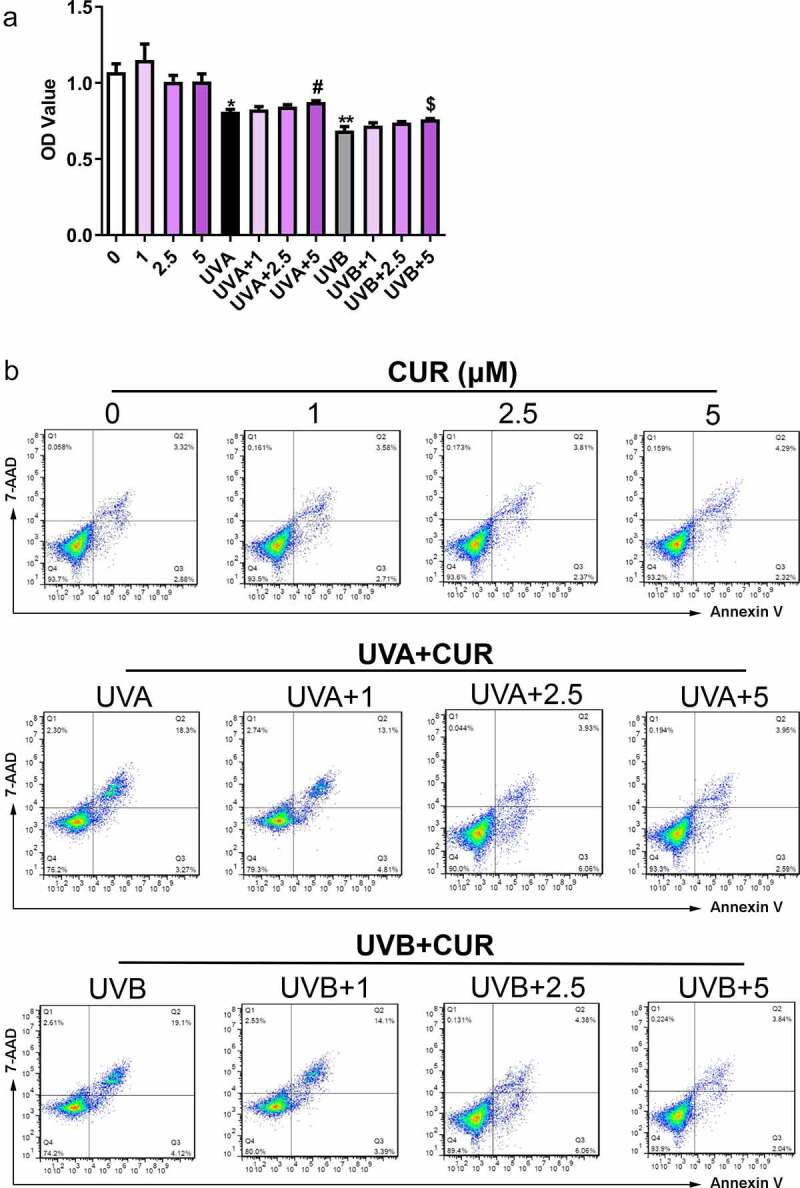
Curcumin reverses the effect induced by UV irradiation. Curcumin could significantly reverse UVA inhibitory effects (or UVB) on cell viability (a) and the promotion effects of UVA (or UVB) on apoptosis and necrosis (b). (UV: ultraviolet; UVA: ultraviolet A; UVB: ultraviolet B; CUR: curcumin; *p < 0.05, **p < 0.01, compared to the control group; ^#^p < 0.05, compared with the UVA group; ^$^p < 0.05, compared with the UVB group)

To select a proper curcumin concentration, Hacat cells were treated with different concentrations (0, 1-μM, 2.5-μM, 5-μM, 10-μM, and 20-μM). The MTS assay showed no difference in cell proliferation when curcumin concentration was 1 μM and 2.5 μM (Figure 2a). In contrast, it was decreased in 5, 10, and 20-μM groups compared with the 0-μM group, in which 5-μM curcumin only decreased a little (Figure 2a). Moreover, a small increase in cell apoptosis was found at the concentration of 20 μM, and the other concentrations had no significant effect on cell apoptosis, indicating that curcumin had no damage to Hacat cells below 20-μM (Figure 2b). As MTS assay showed that 5-μM curcumin had a little decrease in cell proliferation. Flow cell apoptosis analysis using flow cytometry showed that this concentration did not affect Hacat apoptosis (Figure 3b). Therefore, we chose the concentration of 1 μM, 2.5 μM, and 5 μM for the following experiments.

### 3.3 Curcumin attenuates cell proliferation and apoptosis under UV treatments

To study curcumin effects on cell proliferation and apoptosis in UV (UVA in 20-J/cm^2^ and UVB in 57-mJ/cm^2^, respectively) irradiation-induced Hacat cells, the Hacat cells were pretreated with different curcumin concentrations (1, 2.5, and 5-μM) for 24 h, then were treated with UV. MTS results shown in Figure 3a indicate that curcumin (1, 2.5, and 5-μM) did not significantly affect Hacat cell viability (p > 0.05); meanwhile, 20-J/cm^2^ UVA irradiation or 57-mJ/cm^2^ UVB irradiation remarkably suppressed the cell proliferations (p < 0.05); whereas 5-μM curcumin pretreated could effectively reverse the cell damage caused by UV irradiation (p < 0.05). Moreover, compared with the control group, cell apoptosis analysis results indicated that curcumin (1, 2.5, and 5-μM) caused no remarkable damage to Hacat cell, 20-J/cm^2^ UVA irradiation or 57-mJ/cm^2^ UVB irradiation could promote cell apoptosis (the apoptosis rate was 21.5% and 23.2%, respectively), and the apoptosis rate was significantly relieved and pretreated with 5-μM curcumin (the apoptosis rate was 6.5% and 5.9%, respectively) (Figure 3b).

### 3.4 Curcumin induces nuclear accumulation of Nrf2 under UV treatments

**Figure 4.**
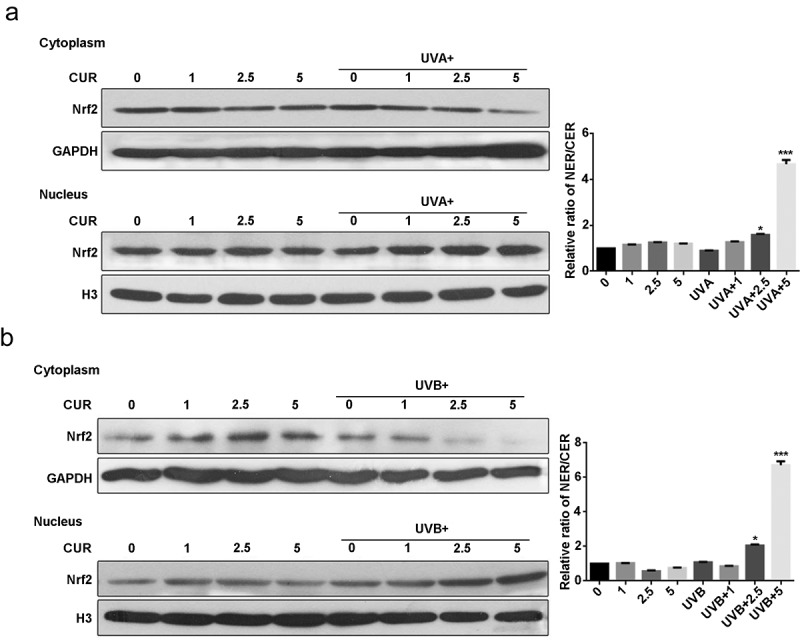
Curcumin induces nuclear accumulation of Nrf2 in Hacat cells. (a) Nrf2 protein expression levels in cytoplasm and nucleus under UVA irradiation with or without being pretreated by curcumin. (b) Nrf2 protein expression levels in cytoplasm and nucleus under UVB irradiation with or without being pretreated by curcumin. (UVA: ultraviolet A; UVB: ultraviolet B; CUR: curcumin; NER: nuclear protein; CER: cytoplasm protein; *p < 0.05, compared to the control group)

Nrf2 protein expression and distribution were analyzed using western blot analysis. As shown in Figure 4, the ratio of the nucleus Nrf2/cytoplasm Nrf2 did not increase under UVA (or UVB) irradiation treatment or curcumin treatment alone; however, pretreatment with curcumin then exposure to UV significantly promoted Nrf2 accumulation in the nucleus of Hacat cells. Moreover, the protein expression of Nrf2 in the nucleus with UVB irradiation was higher than that with UVA irradiation.

### 3.5 Curcumin affects cell antioxidant capacity under UV treatments

**Figure 5.**
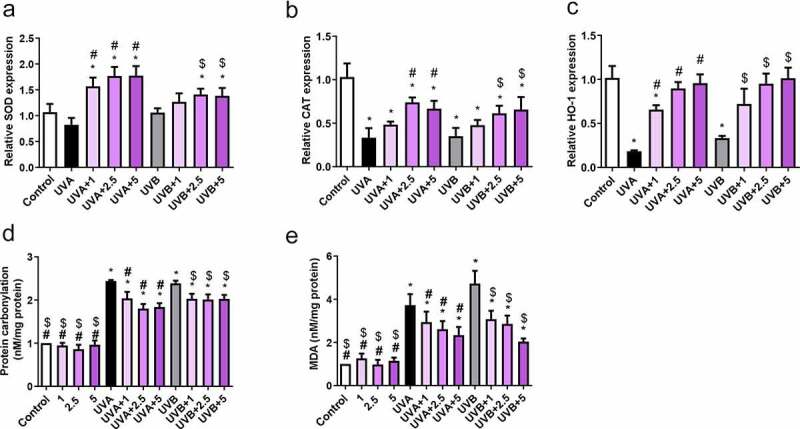
Effects of curcumin on cell antioxidant capacity under UV treatments. Hacat cells were pretreated with indicated curcumin followed the treatment of UVA or UVB. (a) The relative SOD mRNA levels in different groups. (b) The relative CAT mRNA levels in different groups. (c) The relative HO-1 mRNA levels in different groups. (d) The relative protein carbonylation levels in different groups. (e) The relative MDA levels in different groups. (UV: ultraviolet; UVA: ultraviolet A; UVB: ultraviolet B; CAT: catalase; HO-1: heme oxygenase 1; SOD: superoxide dismutase; MDA: carbonylation and malondialdehyde; *p < 0.05 compared to control; ^#^p < 0.05 compared to UVA irradiation; $ p < 0.05 compared to UVB irradiation)

Curcumin and UVA (or UVB) treatments could be potentially implicated in cell antioxidant capacity. Therefore, qPCR was used to examine the mRNA abundance of CAT, HO-1, and SOD. Compared to those in the control group, CAT and HO-1 were downregulated after UVA (20 J/cm^2^) or UVB (57 mJ/cm^2^) irradiation, whereas SOD expression showed no difference (Figure 5a-c). However, curcumin significantly reversed the inhibitory effects of UVA (20 J/cm^2^) or UVB irradiation (57 mJ/cm^2^) on CAT and HO-1 expression, as well as elevated SOD activity (Figure 5a-c). Additionally, protein carbonylation and MDA content in Hacat cells were notably increased after UVA (20 J/cm^2^) or UVB irradiation (57 mJ/cm^2^) compared with the control group; however, it was significantly reduced after being pretreated with curcumin (Figure 5d and 5e). Therefore, 5-μM curcumin was selected for subsequent experiments due to its strong biological activities.

### 3.6 Curcumin protects Hacat cells from acute UV-induced damage via activation of Nrf2

**Figure 6.**
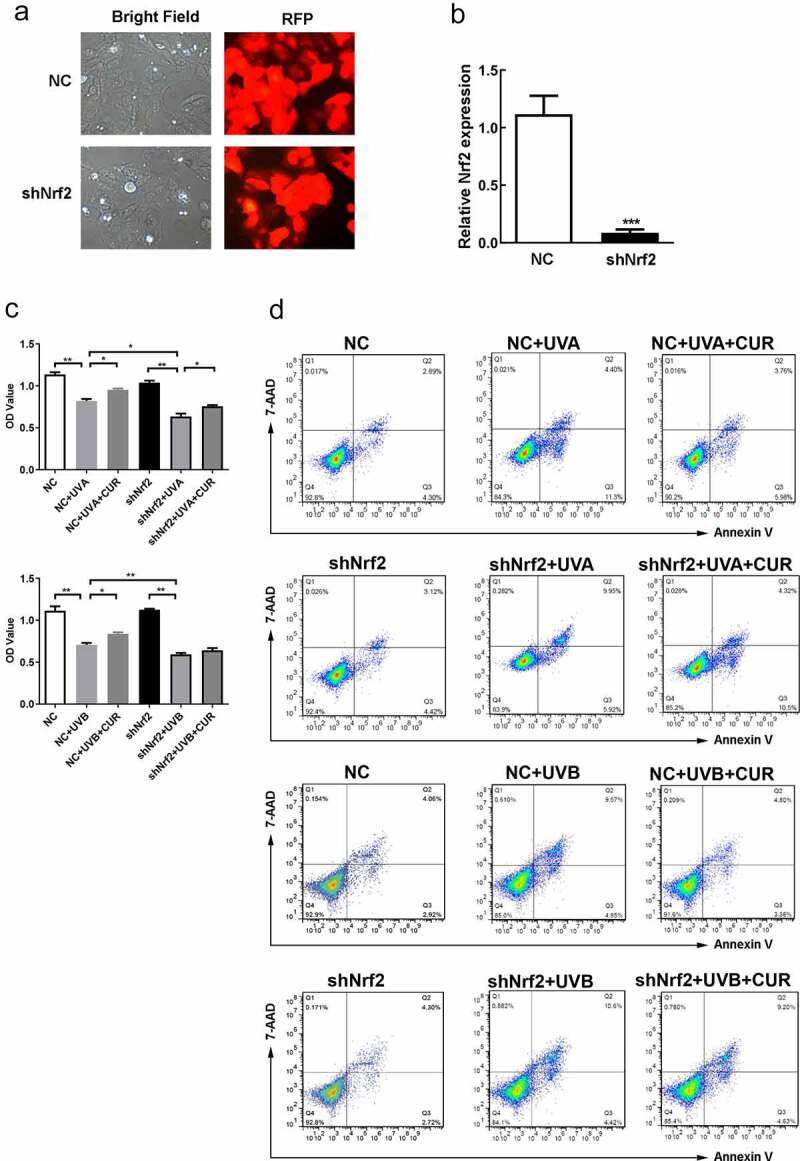
Knockdown of Nrf2 affect Hacat cells vitality and apoptosis under UV and curcumin treatment. (a) The morphology of stable NC Hacat cells and shNrf2 Hacat cells. (b) Detecting the expression of Nrf2 in NC Hacat cells and shNrf2 Hacat cells. (c) The OD values of NC Hacat cells and shNrf2 Hacat cells under UV and curcumin treatment. (d) The apoptosis of NC Hacat cells and shNrf2 Hacat cells under UV and curcumin treatment. (UV: ultraviolet; UVA: ultraviolet A; UVB: ultraviolet B; CUR: curcumin; Nrf2: nuclear factor erythroid 2-related factor 2; RFP: red fluorescent protein; NC: negative control; *p < 0.05, **p < 0.01, ***p < 0.001)

To confirm the role of Nrf2 in curcumin effect on UV-induced damage, we successfully constructed shNrf2 Hacat and NC Hacat as RFP was strong expressed in both cells and the Nrf2 expression decreased in shNrf2 Hacat when compared to NC Hacat (Figure 6a and 6b). The MTS assay showed that the cell viability had no difference between shNrf2 and NC groups (Figure 6c). Furthermore, shNrf2 Hacat and NC Hacat viability were significantly hindered after being exposed to UVA (or UVB), and the inhibition effect in the shNrf2+ UVA (or UVB) group was stronger than that in NC+UVA (or UVB) group (Figure 6c). However, the suppressive effect caused by UVA or UVB irradiation was partially reversed by 5-μM curcumin in NC Hacat but not in shNrf2 Hacat (Figure 6c). Moreover, apoptosis analysis indicated that a significant increase in cell apoptosis was found in NC+UVA (or UVB) and shNrf2+ UVA (or UVB) group; pretreated with 5-μM curcumin partly decreased abnormal cell apoptosis caused by UVA or UVB irradiation in NC Hacat but not in shNrf2 Hacat (Figure 6d). The above results indicate that curcumin reduces damages induced by UVA (or UVB), while interference of Nrf2 expression attenuates the protective effect of curcumin.

**Figure 7.**
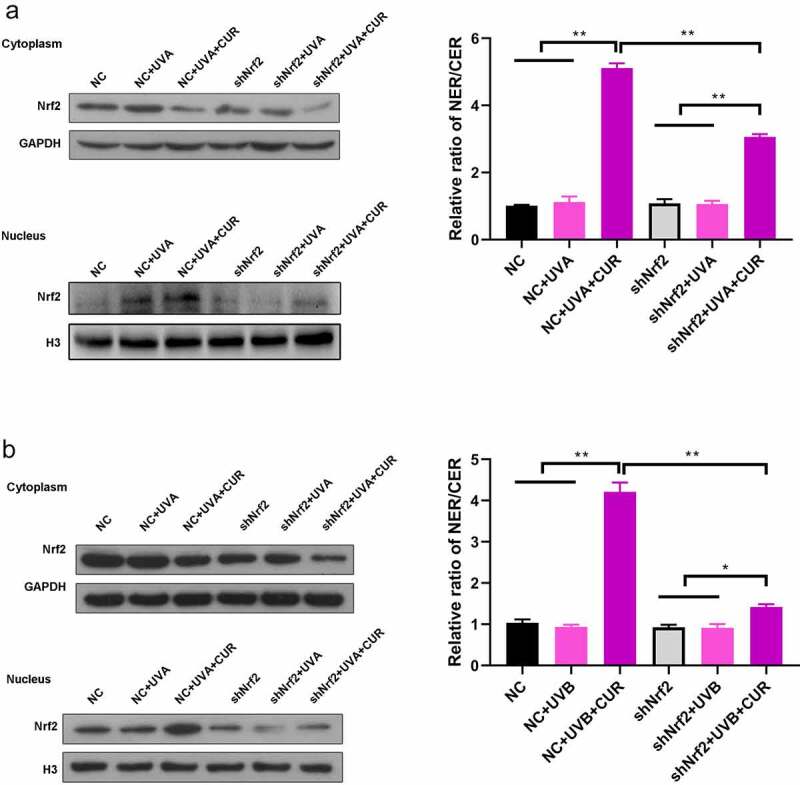
Knockdown of Nrf2 affects the accumulation of Nrf2 in Hacat cells. (a) Nrf2 protein expression levels in cytoplasm and nucleus under UVA irradiation. (b) Nrf2 protein expression levels in cytoplasm and nucleus under UVB irradiation. (UVA: ultraviolet A; UVB: ultraviolet B; CUR: curcumin; Nrf2: nuclear factor erythroid 2-related factor 2; NC: negative control; *p < 0.05, **p < 0.01, NER: nuclear protein; CER: cytoplasm protein)

Subsequently, we evaluated the Nrf2 accumulation in the nucleus. The ratio of nucleus Nrf2/cytoplasm Nrf2 had no change after exposure to UVA (or UVB) irradiation in NC Hacat and shNrf2 Hacat; while pretreatment with 5-μM curcumin significantly increased the ratio of nucleus Nrf2/cytoplasm Nrf2 in NC Hacat and shNrf2 Hacat (Figure 7a and 7b). Moreover, the increased ratio of nucleus Nrf2/cytoplasm Nrf2 was greater in NC Hacat than in shNrf2 Hacat.

**Figure 8.**
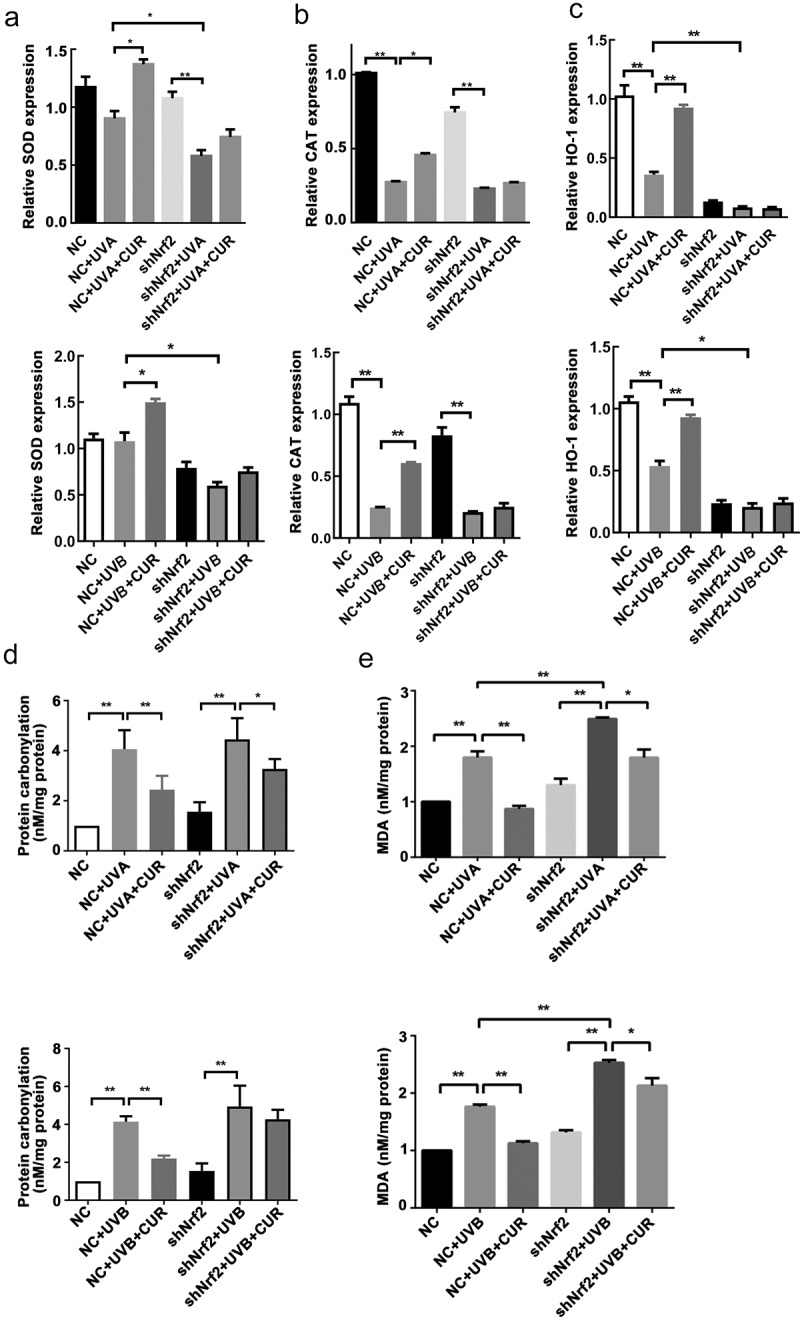
Interference of Nrf2 expression changed the effects of curcumin on cell antioxidant capacity under UV treatments in Hacat cells. (a) The relative SOD mRNA levels in different groups. (b) The relative CAT mRNA levels in different groups. (c) The relative HO-1 mRNA levels in different groups. (d) The protein carbonylation levels in different groups. (e) The MDA levels in different groups. (UV: ultraviolet; UVA: ultraviolet A; UVB: ultraviolet B; CUR: curcumin; Nrf2: nuclear factor erythroid 2-related factor 2; NC: negative control; CAT: catalase; HO-1: heme oxygenase 1; SOD: superoxide dismutase; MDA: carbonylation and malondialdehyde; *p < 0.05, **p < 0.01)

Further analysis was conducted to explore the effects of curcumin on the downstream effector involved in the Nrf2 signaling pathway. After pretreating with or without curcumin followed with UVA (or UVB) exposure, the expression trend of SOD, CAT, HO-1, protein carbonylation, and MDA in NC Hacat cells were similar to in Hacat (Figure 8a-e). While for shNrf2 Hacat cells, the expression of SOD and CAT was significantly decreased, whereas protein carbonylation and MDA expressions were increased after exposure to UVA; CAT expressions was decreased but the expression of protein carbonylation and MDA was increased after exposure to UVB; pretreated with 5-uM curcumin only decreased protein carbonylation and MDA expression in UVA treated cells, and MDA expression was also decreased in UVB treated cells (Figure 8a-e). The above results further confirmed that interference in Nrf2 expression attenuated the protective effect of curcumin on UV-induced Hacat cell damage.

## 4. Discussion

UV radiation was confirmed as the most well-established etiologic factor in skin cancer, including malignant melanoma and non-melanoma. Furthermore, accumulated evidence revealed that UVA permeated deep into the skin and triggered a series of cascade interactions, leading to cellular function destruction and DNA damage [32]. In this study, we explored curcumin’s effect on reversing the damaged Hacat cells induced by UV (UVA and UVB). Therefore, we established a vitro model and detected whether curcumin had inhibitory effects on Hacat cell damage induced by UVA (or UVB) radiation.

Curcumin is widely known for its antioxidant, anti-inflammatory, and antitumor effects and is as safe to humans at therapeutic doses in Phase I trials [33]. Recently, the study executed by Pari L et al showed that curcumin could promote collagen deposition, improve wound healing, and regulate skin disorders [34]. Inspired by the above observation, we hypothesized that curcumin could inhibit UV radiation-induced Hacat cell damage. However, there is a close relationship between UV application time, intensity, and skin damage. In this study, we found that when the UVA irradiation dose reached 20-J/cm^2^, and the UVB irradiation dose reached 57-mJ/cm^2^, the Hacat cells were significantly damaged, which contributed to the morphological change, such as cell deformation, shrink in volume, karyopyknosis and apoptosis. Previous study reports confirmed that the long-term administration of curcumin could cause contact dermatitis [35]. Therefore, we also conducted toleration test with a series of different concerntrations on the Hacat cells. Also, 1-μM, 2.5 μM, and 5-μM were chosen as curcumin reasonable doses for the following experiments.

A study executed by Phillips et al. showed that there was a delay in tumor onset in mice treated with curcumin before or after UVB exposure. And Tsai et al. get a similar observation [36,37]. Curcumin may exert protective effects against UV-induced cytotoxicity in epidermal cells. Consistent with previous studies, this study demonstrated that UV irradiation decreased cell viability and increased apoptosis and necrosis in Hacat cells, while the above effects were reversed with 5-μM curcumin treatment.

Curcumin enhances chemotherapy efficacy by regulating Nrf2 activation [38,39]. Knatko, E. V et al. showed that Nrf2 activation protects against skin carcinogenesis caused by solar-simulated UV radiation [40]. As UVR could not activate Nrf2 effectively, it is not surprising that Nrf2 showed no difference between normal and UVR exposure [41]. It was confirmed that Nrf2 rapidly accumulates in the nucleus under-stimulation, such as antioxidant, and initiates a cascade response to maintain the skin homeostasis. This study discovered that curcumin significantly promoted Nrf2 accumulated in the nucleus of UV-treated Hacat cells, and the Nrf2 nuclear accumulation levels in Hacat cells was higher after UVA irradiation compared to UVB irradiation. When suffering from UV exposure, amounts of malignant and adverse mediators were generated and released into the microenvironment and initiates a cluster of cascade inflammation and immune response. Furthermore, antioxidant cytoprotective proteins, such as SOD, CAT, HO-1 were activated to protect the skin from oxidative stress damage triggered by UV irradiation. In this study, curcumin significantly reversed the downregulated CAT and HO-1 gene expression in Hacat cells after UVA (or UVB) irradiation. Interestingly, SOD expression showed no difference from irradiation alone, while elevated with curcumin treatment after exposing UV irradiation. Such results seemed to hint that curcumin could promote SOD activity independently. Studies have illustrated that polyphenol as bioactive components that could inhibit ROS and other oxidative product accumulation and elevated antioxidant regulators, such as SOD, glutathione peroxidase, and relevant catalase. Therefore, curcumin could promote SOD activity as a bioactive polyphenol compound. Our results also showed that curcumin significantly reversed the up-regulated protein carbonylation and MDA expression in Hacat cells after UVA (or UVB) irradiation, consistent with previous studies [6].

The nuclear translocation of Nrf2 triggered and activated the expression of plentiful useful transcript factors to realize the photoprotection in photoaging. From the above observation, we indicated that the photoprotective effect of curcumin was associated with the modulation of Nrf2-dependent antioxidant response. The effect of Nrf2 was further confirmed by interfering with the Nrf2 expression in Hacat cells. Our results showed that cell viability was significantly decreased in shNrf2 Hacat cells with UV irradiation than in NC Hacat cells. Additionally, 5-μM curcumin treatment partially restored the cell viability compared to UV irradiation treatment alone in NC Hacat cells but not in shNrf2 Hacat cells, which demonstrated that curcumin could decrease the cell injuries, while knockdown Nrf2 attenuated the protective effect of curcumin. Moreover, the cell apoptosis analysis further confirmed this conclusion. Furthermore, the ratio of the nucleus Nrf2/cytoplasm Nrf2 was activated by pretreatment with curcumin followed with UV treatment, which contributed to reverse the decreased expression of SOD, CAT, and HO-1 after UV irradiation. Similarly, the increased protein carbonylation and MDA expression under UV irradiation were also slightly reversed by 5-μM curcumin. However, the silence of Nrf2 attenuated the effect of curcumin. These results revealed that curcumin promoted Nrf2 activation and accelerated the accumulation of antioxidant regulators, which were critical protective molecules for skin screen.

## 5. Conclusion

Altogether, our results demonstrated that the application of curcumin protected against UV irradiation-induced photoaging-associated damage in Hacat cells. The photoprotective effect of curcumin was closely related to the maintenance of antioxidant defenses and inhibition of UV-induced oxidative damage by regulating the Nrf2 signaling pathway in Hacat cells. However, these findings were based only on a vitro model. We will further verify the role of curcumin in animal models, and confirm whether interference of Nrf2 could reverse the protective effect of curcumin.

## Supplementary Material

Supplemental Material

## Data Availability

The data used to support the findings of this study are included within the article.
