# Supplementary material for: Curcumin protection against ultraviolet-induced photo-damage in Hacat cells by regulating nuclear factor erythroid 2-related factor 2
Source: Bioengineered. 2021 Dec 11;12(2):9993–10006. doi: 10.1080/21655979.2021.1994720 (PMC8810050; doi:10.1080/21655979.2021.1994720)
Supplement: Supplemental Material [file KBIE_A_1994720_SM5780.docx]

**Supplemental Table 1. Primers sequence for qPCR**

| Gene | Forward primer (5’-3’) | Reverse primer (5’-3’) |
| --- | --- | --- |
| H-CAT | GAGCCTACGTCCTGAGTCTC | CCGGATGCCATAGTCAGGAT |
| H-HO-1 | AAGACTGCGTTCCTGCTCAAC | AAAGCCCTACAGCAACTGTCG |
| H-SOD | GGAGACTTGGGCAATGTGAC | CACAAGCCAAACGACTTCCA |
| H-Nrf2 | GGATCTGCCAACTACTCCCAG | ACGTAGCCGAAGAAACCTCA |
| H-β-Actin | CATGTACGTTGCTATCCAGGC | CTCCTTAATGTCACGCACGAT |
